# Investigation on outcomes and bacterial distributions of liver cirrhosis patients with gram-negative bacterial bloodstream infection

**DOI:** 10.18632/oncotarget.23582

**Published:** 2017-12-22

**Authors:** Yangxin Xie, Bo Tu, Xin Zhang, Jingfeng Bi, Lei Shi, Peng Zhao, Weiwei Chen, Suxia Liu, Dongping Xu, Enqiang Qin

**Affiliations:** ^1^ Treatment and Research Center for Infectious Diseases, Beijing 302 Hospital, Beijing, China; ^2^ Chinese PLA General Hospital, Medical School, Beijing, China; ^3^ Research Center for Clinical and Translational Medicine, Beijing 302 Hospital, Beijing, China; ^4^ Treatment and Research Center for Liver Failure, Beijing 302 Hospital, Beijing, China

**Keywords:** liver cirrhosis, gram-negative bacterial bloodstream infection, nosocomial infection

## Abstract

**Objective:**

The study aimed at analyzing the epidemiology and outcomes of liver cirrhosis patients undergoing gram-negative bacterial bloodstream infection.

**Results:**

Totally 508 eligible patients were collected, with 25.79% 30-day mortality, and 58.86% patients were confirmed as nosocomial infection. The most common isolates were *Escherichia coli* (48.29%) and *Klebsiella pneumoniae* (19.29%), and multidrug-resistant isolates accounted for 36.61%. The bacterial distributions were similar between survivors and non-survivors (*P*>0.05), but showed close association with acquisition sites of infection (*P*<0.05). Nosocomial infection (HR=1.589, 95% CI=1.004-2.517), Child-Pugh grade (HR=2.471, 95% CI=1.279-4.772), septic shock (HR=1.966, 95% CI=1.228-3.146), complications (HR=3.529, 95% CI=2.140-5.818), and WBC (HR=1.065, 95% CI=1.018-1.114) were independent indicators for 30-day mortality. β-lactamase inhibitor antibiotics exerted a high antibacterial activity.

**Methods:**

The inpatients with liver cirrhosis developed gram-negative bacterial bloodstream infection were collected. The clinical characteristics, bacterial distribution and drug sensitivity results of patients were compared according to their 30-day survival status and acquisition sites of infections. Cox regression model was applied to evaluate the risk factors for 30-day mortality.

**Conclusion:**

*Escherichia coli* and *Klebsiella pneumoniae* are frequently isolated from gram-negative bacterial bloodstream infection episodes in cirrhosis patients. Acquisition site of infection can influence clinical characteristics and etiological distribution. β-lactamase inhibitor antibiotics may be the first choice for empirical treatments.

## INTRODUCTION

Liver cirrhosis is a big threat to people’s health in China. It is well known that China has a high HBV infection rate [1]. According to the statistics, there are about 120 million hepatitis B surface antigen (HBsAg) carriers and nearly 300,000 individuals die from HBV-related liver diseases each year in China [2]. Bloodstream infection (BSI) may be responsible for the high mortality [3]. BSI is one of the serious complications of cirrhosis, representing an important reason for liver failure and death. It has been reported that the occurrence of infection in cirrhosis patients is 10 times more than that in non-cirrhosis individuals [4]. Several risk factors have been confirmed to be connected with the development of BSI in liver cirrhosis patients, such as liver failure, long time of hospital stay, spontaneous bacterial peritonitis (SBP) history, and advanced cirrhosis stage, etc [3, 5]. Timely and appropriate antibiotic treatments are vitally important for prognosis in liver cirrhosis patients developing to BSI [6]. Therefore, it is necessary to investigate the epidemiology of infection and antibiotics sensitivity, thus guiding treatments in liver cirrhosis patients.

The gram-negative bacteria such as *Escherichia coli* and *Klebsiella pneumoniae* are the leading causes for BSI, especially among those hospitalized patients [7]. Gram-negative bacterial infection is a serious challenge in clinic, and its incidence exists differences among different age groups, genders, and populations [8]. At the present time, the third-generation cephalosporin is believed to be the first choice for empirical treatments due to its high antibacterial activity to gram-negative bacteria and good tolerance [9]. However, it is worthy noting that the incidence of multidrug-resistant (MDR) gram-negative bacteria has been increasing in recent years, leading to antibacterial therapy failures and poor outcomes [10–12]. In addition, the distribution of pathogens is distinct in different geographic locations, even in different hospitals located at the same district [13–15]. Therefore, the better knowledge about local epidemiology of gram negative bacteria infection is necessary. However, such a retrospective study with a large sample size is rarely reported in China.

Nosocomial infection is a kind of infection occurring in the hospital environments, such as general wards, ICU, operating rooms, etc, which is resulted from various risk factors, including older age, surgical intervention, and prolonged hospital stays [16]. It has been demonstrated that about 8.7% hospitalized patients may suffer from the infections, and the rate has significantly increased in liver cirrhosis patients admitted to hospital [7, 17]. Previous studies have also reported that the bacterial distribution and antibiotic resistance are significantly different between nosocomial and community-acquired SBP [18]. However, few studies investigate the clinical and microbiological characteristics of gram-negative bacterial infection according to acquisition sites of infections.

In this study, we evaluated the microbiological epidemiology of gram-negative bacterial BSI and potential risk factors for 30-day mortality in liver cirrhosis patients. In addition, the antibiotic susceptibility tests were also performed. The present study was scheduled in Beijing 302 hospital of China, one of the largest infectious disease hospitals in China. Tens of thousands of patients from all over the country are admitted to the hospital for cirrhosis annually. Thus, the results obtained in this study had a certain representation.

## RESULTS

### Baseline characteristics of the included patients

During the study period, 508 eligible patients were enrolled in the current study, including 390 males (76.77%) and 118 females (23.23%), with the average age of 50.96 ± 11.47 years of age. Among these patients, 82 of them (16.14%) were admitted to ICU unit (Table 1).

**Table 1:** Baseline characteristics of the study subjects

Features	Total case (n=508)	Survival status			Acquisition sites of infection		
		Survivors, n=377 (74.21%)	Non-survivors, n=131 (25.79%)	*P*	Nosocomial BSI (n=299, 58.86%)	Community-acquired BSI (n=209, 41.14%)	*P*
**Demographic characteristics**							
Gender				0.203			0.517
Male	390 (76.77)	294 (77.98)	95 (72.52)		232 (77.59)	157 (75.12)	
Female	118 (23.23)	83 (22.02)	36 (37.48)		67 (22.41)	52 (24.88)	
Age (years)	50.96±11.47	50.73±10.96	51.65±12.84	0.021	50.68 ± 11.80	51.37 ± 10.98	0.502
Hospitalization unit				0.000			0.579
General ward	426 (83.86)	350 (92.84)	76 (58.02)		253 (84.62)	173 (82.77)	
ICU	82 (16.14)	27 (7.16)	55 (41.98)		46 (15.38)	36 (17.22)	
**Liver diseases**				0.561			0.171
Single Hepatitis B	329 (64.76)	247 965.52)	82 (62.59)		200 (66.89)	129 (61.72)	
Single Hepatitis C	51 (10.04)	40 (10.61)	11 (8.40)		29 (9.70)	22 (10.53)	
Combined with Hepatitis B and C	8 (1.58)	7 (1.86)	1 (0.76)		37 (12.37)	23 (11.00)	
Alcoholic	60 (11.81)	41 (10.87)	19 (14.50)		6 (2.01)	2 (0.96)	
Others	60 (11.81)	42 (11.14)	18 (13.74)		27 (9.03)	33 (15.79)	
Combined with liver failure (yes, n, %)	119 (23.42)	53 (14.06)	66 (50.38)	0.000	85 (28.43)	34 (16.27)	0.001
Combined with hepatocellular carcinoma (yes, n, %)	141 (27.76)	112 (29.71)	29 (22.14)	0.096	92 (30.77)	49 (23.45)	0.070
**Child-Pugh classification**				0.000			0.020
Class A	46 (9.05)	44 (11.67)	2 (1.53)		35 (11.71)	11 (5.26)	
Class B	168 (33.07)	150 (39.79)	19 (14.50)		103 (34.45)	66 (31.58)	
Class C	293 (57.68)	183 (48.54)	110 (83.97)		161 (53.85)	132 (63.16)	
**BSI data**							
**Infection history within 2 years**				0.263			0.000
Yes	103 (20.28)	72 (19.10)	31 (23.66)		45 (15.05)	58 (27.75)	
No	405 (79.72)	305 (80.90)	100 (76.34)		254 (84.95)	121 (57.89)	
**BSI source**				0.000			0.066
Primary	276 (54.33)	221 (58.62)	55 (41.98)		176 (58.86)	100 (47.85)	
Lung	24 (4.72)	9 (2.39)	15 (11.45)		14 (4.68)	10 (4.78)	
Abdominal (SBP)	204 (40.16)	143 (37.93)	61 (46.56)		106 (34.45)	98 (46.89)	
Urinary tract	4 (0.79)	4 (1.06)	0 (0.00)		3 (1.00)	1 (0.48)	
**Initial presenting symptoms**							
Fever (yes, n, %)	491 (96.65)	368 (97.61)	123 (93.89)	0.041	292 (97.66)	199 (95.21)	0.132
Chilly (yes, n, %)	251 (49.41)	201 (53.32)	50 (38.17)	0.003	157 (52.51)	94 (44.98)	0.095
**Complications**				0.000			0.147
All	362 (71.26)	319 (84.61)	43 (32.82)		215 (71.91)	147 (70.33)	
Upper gastrointestinal bleeding	9 (1.77)	4 (1.06)	5 (3.82)		2 (0.67)	7 (3.35)	
Hepatic encephalopathy	63 (12.40)	39 (10.34)	24 (18.32)		35 (11.71)	28 (13.40)	
Hepato-renal syndrome	27 (5.31)	7 (1.86)	20 (15.27)		19 (6.35)	8 (3.83)	
More than one complication	47 (9.25)	8 (2.12)	39 (29.77)		28 (9.36)	19 (9.09)	
**BSI severity**							
Septic shock				0.000			0.476
yes	110 (21.65)	40 (10.61)	70 (53.43)		68 (22.74)	42 (20.10)	
no	398 (78.35)	337 (89.39)	61 (46.57)		231 (77.26)	167 (79.90)	
**Laboratory data**							
WBC (cells×10^3^/μL)	6.62±5.37	5.82±3.64	8.92±8.19	0.000	5.98 ± 5.58	7.52 ± 4.93	0.001
Serum neutrophil (%)	69.68±16.57	68.68±16.31	72.54±17.05	0.021	0.63 ± 0.15	0.79 ± 0.13	0.000
**Appropriate antibiotics within 12h**				0.008			0.016
Yes	367 (72.24)	284 (75.33)	83 (63.36)		204 (68.23)	163 (77.99)	
No	141 (27.76)	93 (24.67)	48 (36.64)		95 (31.77)	46 (22.10)	
Nosocomial infection (yes, n, %)	299 (58.86)	207 (54.91)	92 (70.23)	0.002	-	-	-
30-day survival (survivors, 1, %)	377 (74.21)	-	-	-	207 (69.23)	170 (81.34)	0.002

Notes: ICU, Intensive care unit; BSI, Bloodstream infection; WBC, White blood cell; -, indicated no related data.

HBV was the most common reason for liver cirrhosis, which accounted for 64.76% and was followed by alcohol (11.81%) and hepatitis C (10.04%). Some of the patients presented liver failure (23.42%) and other patients (27.76%) were diagnosed with cirrhosis combined with hepatocellular carcinoma. According to Child-Pugh score, 9.05% patients were grouped to Class A and 33.07% patients were grouped to Class B, while over half of the patients (57.68%) were confirmed as Class C (Table 1).

The baseline information of BSI is summarized in Table 1. More than half of the patients (58.86%) were confirmed as nosocomial infection, and 20.28% patients were with infection history less than 2 years. Primary infection was the main source for BSI (accounting for 54.33%), and SBP was the second source for infection (accounting for 40.16%). Other infection sources included lung (4.72%) and urinary tract (0.79%). Fever was the most frequently observed symptom and 96.65% patients initially presented this symptom. Chilly occurred in 49.41% patients. Some complications were observed in the enrolled patients, containing hepatic encephalopathy (12.40%) and hepato-renal syndrome (5.31%). Furthermore, 9.25% patients were diagnosed with more than one complications, and 21.65% patients presented with septic shock (Table 1).

In addition, laboratory data suggested that the average WBC of eligible patients was 6.62 ± 5.37 (cells×10^3^/μL), while serum neutrophil was 69.68% ± 16.57%. Results *in vitro* experiments proved that 72.24% patients received appropriate antibiotics within 12h (Table 1).

### Clinical characteristics of survivors and non-survivors

The primary outcomes of enrolled patients were evaluated by using the 30-mortality. In the present study, 131 cirrhosis patients (25.79%) died within 30 days after BSI diagnosis. The clinical characteristics were compared between survivors and non-survivors. The results of analyses indicated that age, ICU admission, combined with liver failure, Child-Pugh score, the rate of nosocomial infection, BSI source, occurrence of fever and chilly, complications, septic shock, and laboratory data were significantly different between survivors and no-survivors (*P*<0.05 for all). Furthermore, patients receiving appropriate antibiotics within 12h after BSI onset had a higher survival rate than those who were treated with impertinent antibiotics (*P*=0.008). In addition, the gender rate, types of liver disease, and occurrence of hepatocellular carcinoma were similar between survivors and non-survivors (*P*>0.05 for all) (Table 1).

### Comparison of clinical characteristics between nosocomial and community-acquired infection

Among the patients, 299 of them (58.86%) were diagnosed with nosocomial BSI. The effects of acquisition sites of infection on clinical characteristics were investigated in the current study. Results demonstrated that Child-Pugh grade (*P*=0.020), liver failure (*P*=0.001), infection history within 2 years (*P*=0.000), WBC (*P*=0.001) and serum neutrophil (*P*=0.000) were obviously different between nosocomial infection and community-acquired infection. Moreover, nosocomial infection was significantly correlated with inappropriate antibiotics within 12h (*P*=0.016) and high 30-day mortality (*P*=0.002) (Table 1).

### Bacterial distributions of the survivors and non-survivors

In our early study [19], *Escherichia coli* was the most common pathogen, which accounted for 48.23% and was followed by *Klebsiella pneumoniae* (19.29%). Some other common isolates included *Aeromomas species* (5.91%), *Enterobacter cloacae* (3.74%), *Acinetobacter baumanii* (3.15%), *Pseudomonas aeruginosa* (1.77%), *Stenotrophomonas maltophilia* (1.18%), and others (14.76%). Additionally, 1.97% patients were proved to be infected by more than one pathogenic bacteria (Table 2).

**Table 2:** The distribution of bacteria among the study population

	Total, n=508	Survival status			Acquisition sites of infection		
		Survivors, n=377 (74.21%)	Non-survivors, n=131 (25.79%)	*P*	Nosocomial BSI, n=299 (58.86%)	Community-acquired BSI, n=209 (41.14%)	*P*
Bacterial distributions							
*Escherichia coli*	245 (48.23)	185 (49.07)	60 (45.80)	0.519	135 (45.15)	110 (52.63)	0.097
*Klebsiella pneumoniae*	98 (19.29)	72 (19.10)	26 (19.85)	0.851	49 (16.39)	49 (23.45)	0.047
*Pseudomonas aeruginosa*	9 (1.77)	5 (1.33)	4 (3.05)	0.197	9 (3.01)	0 (0.00)	0.011
*Enterobacter cloacae*	19 (3.74)	14 (3.71)	5 (3.82)	0.957	16 (5.35)	3 (1.43)	0.022
*Aeromonas species*	30 (5.91)	24 (6.37)	6 (4.58)	0.455	15 (5.02)	15 (7.18)	0.309
*Acinetobacter baumanii*	16 (3.15)	6 (1.59)	10 (7.63)	0.001	14 (4.68)	2 (0.96)	0.018
*Stenotrophomonas maltophilia*	6 (1.18)	3 (0.80)	3 (2.29)	0.173	6 (2.01)	0 (0.00)	0.039
Others	75 (14.76)	61 (16.18)	14 (10.69)	0.127	52 (17.39)	23 (11.00)	0.046
Mixed	10 (1.97)	7 (1.86)	3 (2.29)	0.758	3 (1.00)	7 (3.35)	0.061
ESBL status of the isolated pathogens				0.061			0.302
ESBL (+)	138 (39.09)	97 (36.33)	41 (47.67)		79 (41.58)	59 (36.92)	
ESBL (-)	215 (60.91)	170 (63.67)	45 (52.33)		111 (58.42)	104 (63.80)	

Notes: Mixed, meant infected by more than one bacteria; ESBL, Extended-spectrum β-lactamase.

ESBL statuses of *Escherichia coli* and *Klebsiella pneumoniae* were evaluated in the current study. Approximately 40.12% of *Escherichia coli* and *Klebsiella pneumoniae* presented positive ESBL and the rest (59.88%) was negative (Table 2).

We compared the epidemiological distributions between survivors and non-survivors. The results suggested that the distribution of *Acinetobacter baumanii* was significantly different between survivors and non-survivors (*P*=0.001), while the occurrence rate of other pathogens was similar between survivors and non-survivors (*P*>0.05 for all). In addition, the distribution of ESBL producing pathogens was also similar between survivors and non-survivors (*P*=0.061) (Table 2).

### Comparison of epidemiological distribution between nosocomial BSI and community-acquired BSI

Chi-square test was applied to compare the distribution of pathogens between nosocomial BSI and community-acquired BSI. The results showed that the distributions of *Klebsiella pneumoniae* (*P*=0.047), *Pseudomonas aeruginosa* (*P*=0.011), *Enterobacter cloacae* (*P*=0.022), *Acinetobacter baumanii* (*P*=0.018), *Stenotrophomonas maltophilia* (*P*=0.039) and others (*P*=0.046) were markedly different between patients diagnosed with nosocomial BSI and those who were confirmed to be community acquired BSI. However, the occurrence rates of *Escherichia coli*, *Aeromonas species*, and mixed were similar between the comparable groups (*P*>0.05 for all). Additionally, the distribution of ESBL producing pathogens was similar between BSI acquired from different sites (Table 2).

### Drug sensitivity analysis

Drug sensitivity test was carried out in the current study. About 91.65% isolates were sensitive to Piperacillin/tazobactam. Compared with Piperacillin/tazobactam, the isolated pathogens were more sensitive to carbapenems, with an average sensitive rate of 96.025%, and Amikacin (96.62%) (*P*<0.05 for both). In addition, the sensitive rates of cefepime (75.97%), cefoperazone (68.10%), cefotaxime (64.65%), ceftazidime (73.33%), ceftriaxone (63.19%), gatifloxacin (71.50%), and levofloxacin (70.25) were significantly lower than that of Piperacillin/tazobactam (*P*<0.05 for all) (Table 3).

**Table 3:** Drug sensitivity analysis

Antibiotics	Total	Sensitivity (n, %)	*P* value
The fourth-generation cephalosporins			
Cefepime	462	351 (75.97)	0.000
**The third-generation cephalosporins**			
Cefoperazone	163	111 (68.10)	0.000
Cefotaxime	198	128 (64.65)	0.000
Ceftazidime	480	352 (73.33)	0.000
Ceftriaxone	470	297 (63.19)	0.000
**Quinolones**			
Gatifloxacin	200	143 (71.50)	0.000
Levofloxacin	474	333 (70.25)	0.000
**Carbapenems**			
Imipenem	485	464 (95.67)	0.000
Meropenem	387	373 (96.38)	0.000
**β-lactamase inhibitors**			
Piperacillin/tazobactam	467	428 (91.65)	**Reference**
Cefperazone/sulbactam	405	364 (89.88)	0.117
**Aminoglycosides**			
Amikacin	473	457 (96.62)	0.000

**Figure 1:**
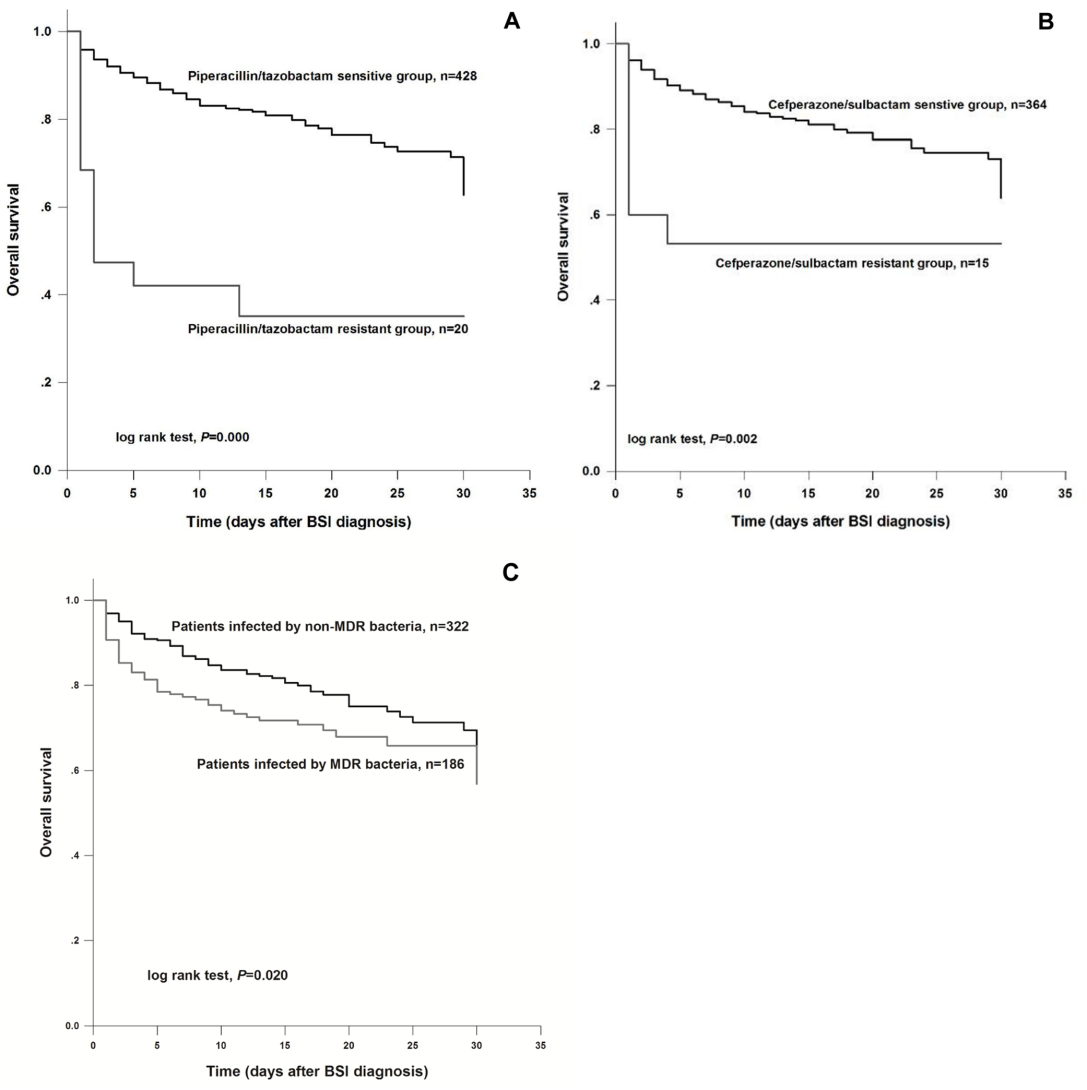
(A) Overall survival analysis for liver cirrhosis patients according to their susceptibility to piperacillin/tazobactam The results suggested that patients with drug resistance had a poor overall survival than those sensitive to piperacillin/tazobactam (log rank test, *P*=0.000). **(B)** Survival curve for the study subjects according to their resistance to cefperazone/sulbactam. The curve showed that patients carrying cefperazone/sulbactam resistant pathogen underwent a lower survival rate than those carrying sensitive bacteria (log rank test, *P*=0.002). **(C)** Survival analysis for the cirrhosis cases infected by MDR. Analysis results demonstrated that the survival rate of liver cirrhosis cases infected by MDR bacteria was significantly low, compared with those infected by non-MDR bacteria (log rank test, *P*=0.020).

Additionally, the overall survival analyses were carried out for recruited patients in accordance with the β-lactamase inhibitors resistance of their isolated pathogens. Survival curves demonstrated that patients infected by piperacllin/tazobactam or cefperazone/sulbactam resistant pathogens had a significantly poor survival rate (log rank test, *P*<0.05 for both) (Figures 1A and 1B).

### Drug sensitivity analysis between nosocimial BSI and community-acquired BSI

In the present study, we compared antibiotics sensitivity between pathogens isolated from nosocomial BSI and other sites. The results demonstrated that the pathogens isolated from hospital-acquired BSI had a higher resistant rate to carbapenems than those isolated from community-acquired BSI (*P*<0.05). However, the drug resistances of other antibiotics were not influenced by the sites of infection (*P*>0.05 for all) (Table 4).

**Table 4:** The comparison of drug sensitivity between nosocomial BSI and community-acquired BSI

	Total	Nosocomial BSI	Community-acquired BSI	*P* value
The fourth-generation cephalosporins				
Cefepime				0.300
Sensitive	351 (75.97)	195 (73.58)	156 (79.19)	
Mid-sensitive	11 (2.38)	8 (3.02)	3 (1.52)	
Resistant	100 (21.65)	62 (23.40)	38 (19.29)	
**The third-generation cephalosporins**				
Cefoperazone				0.170
Sensitive	111 (68.10)	56 (62.22)	55 (75.34)	
Mid-sensitive	8 (4.91)	6 (6.67)	2 (2.74)	
Resistant	44 (26.99)	28 (31.11)	16 (21.92)	
Cefotaxime				0.600
Sensitive	128 (64.65)	76 (64.96)	52 (64.20)	
Mid-sensitive	8 (4.04)	6 (5.13)	2 (2.47)	
Resistant	62 (31.31)	35 (29.91)	27 (33.33)	
Ceftazidime				0.531
Sensitive	352 (73.33)	202 (71.63)	150 (75.76)	
Mid-sensitive	10 (2.08)	7 (2.48)	3 (1.51)	
Resistant	118 (24.58)	73 (25.89)	45 (22.73)	
Ceftriaxone				0.655
Sensitive	297 (63.19)	165 (61.57)	132 (65.35)	
Mid-sensitive	14 (2.98)	9 (3.36)	5 (2.47)	
Resistant	159 (33.83)	94 (35.07)	65 (32.18)	
**Quinolones**				
Gatifloxacin				0.928
Sensitive	143 (71.50)	84 (71.19)	59 (71.95)	
Mid-sensitive	6 (3.00)	4 (3.39)	2 (2.44)	
Resistant	51 (25.50)	30 (25.42)	21 (25.61)	
Levofloxacin				0.790
Sensitive	333 (70.25)	192 (69.31)	141 (71.57)	
Mid-sensitive	18 (3.80)	10 (3.61)	8 (4.06)	
Resistant	123 (25.95)	75 (27.08)	48 (24.36)	
**Carbapenems**				
Imipenem				0.036
Sensitive	464 (95.67)	265 (93.97)	199 (98.03)	
Mid-sensitive	4 (0.83)	2 (0.71)	2 (0.98)	
Resistant	17 (3.50)	15 (5.32)	2 (0.98)	
Meropenem				0.038
Sensitive	373 (96.38)	205 (94.91)	168 (98.25)	
Mid-sensitive	6 (1.55)	3 (1.39)	3 (1.75)	
Resistant	8 (2.07)	8 (3.70)	0 (0.00)	
**β-lactamase inhibitors**				
Piperacillin/tazobactam				0.263
Sensitive	428 (91.65)	245 (90.07)	183 (93.85)	
Mid-sensitive	19 (4.07)	12 (4.41)	7 (3.59)	
Resistant	20 (4.28)	15 (5.51)	5 (2.56)	
Cefperazone/sulbactam				0.089
Sensitive	364 (89.88)	200 (87.34)	164 (93.18)	
Mid-sensitive	26 (6.42)	20 (8.73)	6 (3.41)	
Resistant	15 (3.70)	9 (3.93)	6 (3.41)	
**Aminoglycoside**				
Amikacin				0.317
Sensitive	457 (96.62)	261 (95.60)	196 (98.00)	
Mid-sensitive	6 (1.27)	5 (1.83)	1 (0.50)	
Resistant	10 (2.11)	7 (2.56)	3 (1.50)	

### Association between MDR bacteria infection and clinical characteristics in liver cirrhosis patients

According to the drug sensitivity analysis, 186 isolates were defined as MDR bacteria, and the percentage of MDR isolates was 36.61%. In the current study, all the ESBL positive bacteria were MDR bacteria. We compared the clinical symptoms between patients infected by MDR bacteria and those infected by non-MDR bacteria. The results listed in Table 5 suggested that MDR bacteria infection was significantly correlated with elder age (*P*=0.044), ICU admission (*P*=0.003), infection history within 2 years (*P*=0.033), and advanced Child-Pugh grade (*P*=0.034). Furthermore, the patients infected by MDR bacteria were more likely to undergo septic shock (*P*=0.000), inappropriate antibitics within 12h (*P*=0.000), and poor survival within 30 days (*P*=0.020) (Table 5). In addition, survival analysis suggested that the patients infected by MDR bacteria had poor survival, compared with those infected by non-MDR bacteria (log rank test, *P*=0.020) (Figure 1C).

**Table 5:** The association between MDR BSI infection and clinical characteristics in liver cirrhosis patients

Factors	MDR infection (n=186, 36.61%)	Non-MDR infection (n=322, 63.39%)	*P* values
Gender			0.067
male	134 (72.04)	255 (79.19)	
female	52 (27.96)	37 (11.49)	
Age (years)	52.31±11.55	50.19±11.36	0.044
ICU			0.003
yes	42(22.58)	40 (12.42)	
no	144 (77.42)	282 (87.58)	
Nosocomial infection			0.301
yes	115 (61.83)	184 (57.14)	
no	71 (38.17)	138 (42.86)	
Infection history within 2 years			0.033
yes	47 (25.27)	56 (17.39)	
no	139 (74.73)	266 (82.61)	
SBP as BSI source			0.170
yes	82 (44.09)	122 (37.89)	
no	104 (55.91)	200 (62.11)	
Child-Pugh grade			0.034
A+B	67 (36.02)	147 (45.65)	
C	119 (63.98)	175 (54.35)	
Combined with liver failures			0.456
yes	47 (25.27)	72 (22.36)	
no	139 (74.73)	250 (77.64)	
Combined with liver cancer			0.341
yes	47 (25.27)	94 (29.19)	
no	139 (74.73)	228 (70.81)	
Septic shock			0.000
yes	56 (30.11)	54 (16.77)	
no	130 (69.89)	268 (83.23)	
Complications			0.082
yes	124 (66.67)	238 (73.91)	
no	62 (33.33)	84 (26.09)	
WBC (cells×10^3^/μL)	7.19±6.83	6.28±4.28	0.065
Serum neutrophil (%)	0.71±0.15	0.69±0.17	0.095
Appropriate antibiotics within 12h			0.000
yes	104 (55.91)	263 (81.68)	
no	82 (44.09)	59 (18.32)	
30-day survival status			0.020
survivor	127 (68.28)	250 (77.64)	
non-survivor	59 (31.72)	72 (22.36)	
ESBL			0.000
positive	138	0	
negative	11	204	

### Risk factors for 30-day mortality in gram-negative bacterial bloodstream infection patients

Cox regression model was applied to evaluate the risk factors for 30-mortality of patients. Through the univariate analyses, the ICU admission, nosocomial infection, Child-Pugh grade, combined with liver failure, septic shock, complications, WBC, serum neutrophil, appropriate antibiotics within 12h and MDR bacteria infection were reported to be associated with 30-day mortality among cirrhosis patients developing to BSI (*P*<0.05 for all). Multivariate analyses suggested that nosocomial infection (HR=1.589, 95% CI=1.004-2.517, *P*=0.048) Child-Pugh grade (HR=2.471, 95% CI=1.279-4.772, *P*=0.007), septic shock (HR=1.966, 95% CI=1.228-3.146, *P*=0.005), complications (HR=3.529, 95% CI=2.140-5.818, *P*=0.000), and WBC (HR=1.065, 95% CI=1.018-1.114, *P*=0.006) were independent indicators for 30-day mortality among the study population (Table 6).

**Table 6:** Risk factors for 30-mortality in liver cirrhosis patients infected with gram-negative bacteria

Factors	Univariate analyses		Multivariate analyses	
	HR (95%CI)	*P*	HR (95%CI)	*P*
Gender (male vs female)	0.770 (0.525-1.130)	0.182		
Age (years)	1.010 (0.995-1.026)	0.207		
ICU (yes vs no)	4.280 (3.021-6.065)	0.000		
Nosocomial infection (yes vs no)	1.724 (1.185-2.508)	0.004	1.589 (1.004-2.517)	0.048
Infection history within 2 years (yes vs no)	1.198 (0.801-1.793)	0.379		
SBP as BSI source (yes vs no)	1.258 (0.892-1.774)	0.191		
Child-Pugh grade (C vs A+B)	4.017 (2.492-6.475)	0.000	2.471 (1.279-4.772)	0.007
Combined with liver failures (yes vs no)	3.244 (2.297-4.579)	0.000		
Combined with liver cancer (yes vs no)	1.251 (0.832-1.882)	0.282		
Septic shock (yes vs no)	5.454 (3.864-7.698)	0.000	1.966 (1.228-3.146)	0.005
Complications (yes vs no)	6.444 (4.461-9.310)	0.000	3.529 (2.140-5.818)	0.000
WBC (cells×10^3^/μL)	1.084 (1.060-1.109)	0.000	1.065 (1.018-1.114)	0.006
Serum neutrophil (%)	4.188 (1.354-12.960)	0.013		
Appropriate antibiotics within 12h (no vs yes)	1.575 (1.104-2.248)	0.012		
ESBL status (positive vs negative)	1.443 (0.945-2.203)	0.090		
MDR bacteria infection (yes vs no)	1.539 (1.091-2.172)	0.014		

Notes: BSI, Bloodstream infection; ICU, Intensive care unit; SBP, Spontaneous bacterial peritonitis; WBC, White blood cell; ESBL, Extended-spectrum β-lactamase.

## DISCUSSION

BSI is one of the serious complications of liver cirrhosis, with high morbidity and mortality rates. Compared with non-cirrhosis patients, the occurrence of BSI is significantly high in liver cirrhosis. The underlying mechanisms may refer to two pathophysiological conditions: dysregulated intestinal bacterial translocation and immune dysfunction caused by cirrhosis. Patients with liver cirrhosis exhibit slow peristalsis of intestine and congestion of the gastrointestinal tract, which create a suitable condition for growth of pathogens. Moreover, the surveillance function of liver against bacteria is weaken in the cirrhosis cases. The interaction between the two pathophysiological conditions may lead to excessive growth of intestinal flora, thus causing the occurrence of BSI. Although the managements of cirrhosis have been improved, the incidence rate of BSI is still high [19]. Once the infection and multiple complications such as septic shock occur, it will be followed by organ failures. Thus, timely and appropriate antibiotic treatment is important for improving quality of life and outcomes in cirrhosis patients combined with BSI. The present study was carried out to evaluate the etiology of gram-negative bacterial infection in cirrhosis patients, as well as the antibiotic sensitivity of their isolates, which might guide empirical therapy.

In the present study, 30-day mortality was used to measure the primary outcomes of liver cirrhosis patients and the general clinical characteristics were compared between survivors and no-survivors. The results revealed that various factors including Child-Pugh score, BSI data, age, and therapeutic regimens were different between survivors and non-survivors. Child-Pugh score as a liver disease specific score was widely used to predict long and short term survival in cirrhosis patients, even among those combined with infection [20]. However, it was reported that besides the general scoring systems, the infection severity should also be considered when predicting prognosis in liver cirrhosis patients presenting infection [21]. In this study, we found that in addition to Child-Pugh score, septic shock, complications, WBC and hospital-acquired BSI were also independently correlated with 30-day survival rate among cirrhosis patients infected by gram negative bacteria. The conclusion informed that both liver diseases and infection played important roles in prognosis of cirrhosis patients who presented gram negative bacterial infection. Therefore, a novel model, which could predict liver disease and infection, was urgently needed for prognosis analysis in the study population.

The etiological distribution of cirrhosis patients was reported in a number of studies. In the study designed by Campillo et al., gram-negative and positive bacteria as well as *Candida albicans* are cultured from the specimens of hospital-acquired infection patients [22]. A study carried out by Bartoletti et al. reports that the major cause for BSI is gram-negative bacteria, followed by gram-positive bacteria and *Candida albicans* [23]. However, a specific study on gram negative bacterial infection is absent, especially in China. In the current study, cirrhosis patients infected with gram negative bacteria were employed as study subjects. Results of analyses stated that *Escherichia coli* and *Klebsiella pneumoniae* were the major causes for gram-negative bacterial infection. The microbiological etiology was similar between survivors and non-survivors. The conclusions were consistent with the study performed by Bartoletti et al. [23].

Nosocomial infection was proved to be a risk factor for short and long-term mortality in cirrhosis patients developing to BSI [24]. Bert et al. reported that the microbiological distribution and therapeutic complications were significantly different between nosocomial BSI and community-acquired BSI [18]. However, the therapeutic regimens were similar between nosocomial and community-acquired BSI, which might be responsible for the high mortality in hospital-acquired BSI patients. In the present study, we found that the cirrhosis patients with advanced Child-Pugh grade, presenting liver failure, high WBC and serum neutrophil, or having infection history less than 2 years were more likely develop to nosocomial BSI. Moreover, the nosocomial infection was observed to be correlated with high mortality and inappropriate antibiotic regimens within 12h. All these results implied that nosocomial infection could aggravate the disease progression of liver cirrhosis, thus leading to limited therapeutic efficacy and dismal clinical outcomes. In addition, the acquisition sites of infection was adopted to compare the bacterial distribution and drug sensitivity. The distributions of *Klebsiella pneumoniae*, *Pseudomonas aeruginosa*, *Enterobacter cloacae*, *Acinetobacter baumanii*, and *Stenotrophomonas maltophilia* were markedly different between nosocomial and community-acquired gram-negative BSI. Furthermore, cox regression analysis suggested that nosocomial infection was an independent biomarker for 30-mortality of the collected patients. All the related results revealed that acquisition sits of infection could influence the outcomes of cirrhosis patients combined with gram-negative bacterial infection. A study scheduled by Cheong et al. proved that the bacterial distribution and outcomes were obviously different between nosocomial and community infection, which was consistent with our findings [25]. They also have discovered that third-generation cephalosporins sensitivities in hospital- and community- acquired BSI were different. However, drug sensitivity analyses in the current study demonstrated that except for carbapenems, antibiotic sensitivities were similar between nosocomial and community-acquired infection. Various factors including different hospitals and study populations might contribute to the differences. Therefore, a multicenters study was needed to evaluate the effects of nosocomial infection in cirrhosis patients.

Antibiotic management was crucially important for outcomes in cirrhosis patients combined with infection. Unfortunately, with the increasing drug resistant bacteria, anti-bacteria treatments became difficult [26]. In the current study, 36.61% isolates were MDR bacteria. MDR bacteria were frequently isolated from elder patients, ICU cases, and those diagnosed with advanced Child-Pugh grade. Furthermore, liver cirrhosis cases infected by MDR pathogens were more likely to undergo inappropriate antibiotic treatments and unsatisfactory clinical outcomes. Therefore, a better understanding of the antibiotic sensitivity is key for infection treatments. In the present study, the sensitivity of frequently-used antibiotics based on gram-negative bacteria isolated from the study population was compared, and the results demonstrated that the isolates showed high sensitivity to carbapenem, aminoglycoside, and β-lactamase inhibitor antibiotics. Compared with β-lactamase inhibitors antibiotics, the drug sensitivities of carbapenem and aminoglycosides were significantly high, while other antibiotics such as cephalosporins, quinolones exhibited low antibacterial activity. Aminoglycosides antibiotics were frequently used for the infection caused by aerobic gram negative bacteria, but the nephrotoxicity, ototoxicity and drug resistance might limit its wide use [27, 28]. At the present time, carbapenems represented a last line for treatments of multidrugs resisting gram-negative pathogens, which was often used in empirical therapy [29]. However, growing evidences had proved that the excessive use of carbapenems could promote the prevalence of carbapenems resistant pathogens [30, 31]. Thus, an effective antibiotic which could serve as carbapenems alternatives in treatment of infection was urgently needed. In this study, the β-lactamase inhibitor antibiotics showed a high antibacterial activity to gram-negative pathogens. A meta-analysis conducted by Shiber et al. have demonstrated that there were no difference in efficacy between β-lactamase inhibitors and carbapenms in treatment of infections. Moreover, no serious side effects were observed in β-lactamase inhibitor treatments [32]. All of the related data revealed that β-lactamase inhibitor antibiotics showed a high antibacterial activity without serious side effects, which might be the first choice for empirical treatments of gram-negative bacterial infection.

There were still several limitations in the current study. First, we only investigated the microbiological characteristics and clinical outcomes of gram-negative bacterial infection cases. However, growing evidences have suggested that the prevalence of the gram-positive bacteria like *Staphylococcus* exhibited obviously increased trend in BSI cases during the last 10 years, due to the increased application of invasive procedures and exogenous route of infection [33]. Therefore, it is necessary to collect more liver cirrhosis combined BSI cases to roundly investigate the microbiological etiology of BSI in Chinese population. Second, we did not investigate the pharmacodynamics characteristics of β-lactamase inhibitor antibiotics for treatment BSI in the current study. The conditions of liver cirrhosis patients developing BSI are often critical, and appropriate empirical treatments are pivotal for therapeutic effects. The study scheduled by Zelenitsky et al. reported that for ICU patients presenting infection, piperacillin-tazobactam 3.375 g every 6h, with prolonged infusion times of 0.5 h could significantly control infection condition [34]. Ambrose et al. found that for patients infected by *E. coli*, the optimal regimen of piperacillin-tazobactam was infusion with 3.375 g every 4 h, and 3.375 g every 6 h for *K. pneumoniae* infection [35]. The prolonged infusion is necessary for critically ill patients [36]. A retrospective cohort study carried out by Yost et al. indicated that extended-infusion of piperacillin-tazobactam could significantly improve the survival and lower the mortality among patients infected by gram-negative bacteria [37]. The administration experiences might provide guidance for application of β-lactamase inhibitor antibiotics in clinic. In addition, cox analysis was used to identify the risk factors of 30-day mortality among the study population. The clinical factors were detected when BSI onset. However, some of the parameters, such as WBC, complications, septic shock, might change during the disease progression, thereby causing bias to the final results. In a word, further investigations are still required to address the above issues.

In conclusion, *Escherichia coli* and *Klebsiella pneumoniae* are the prevalent isolates from gram-negative bacterial bloodstream infection episodes in cirrhosis patients. The clinical characteristics and etiological distribution may be influenced by the acquisition sites of infection. Nosocomial infection, Child-Pugh grade, septic shock, complications and WBC are independent biomarkers for 30-mortality in the study populations. β-lactamase inhibitor antibiotics show a high antibacterial activity, which may be widely used in empirical therapy.

## MATERIALS AND METHODS

### Study subjects

The present research was a retrospective cohort study. The patients who were hospitalized for liver cirrhosis and developed to gram-negative bacterial bloodstream infection in Beijing 302 hospital from October, 2010 to January, 2015 were recruited in our study. The present study was based on adult group and the patients aged over 16 were collected. In addition, the clinical information of recruited patients was extracted from the medical records, such as demographic data, hospitalization information, complications, BSI data, microbiological testing results, and laboratory data. In those patients who developed multiple BSIs during hospital duration, only the first episode was used for analysis. The current study was approved by the ethic committee of the hospital. All of the patients or their families signed the informed consents.

### Data collection

Data were extracted from the clinical records of eligible patients using a standardized data form. The following information was collected: demographic characteristics (gender and age), hospitalization unit, cause of cirrhosis, Child-Pugh score, BSI data (history within 2 years, BSI source, days for hospitalization before BSI onset, initial symptoms, complications, septic shock), laboratory data (WBC, serum neutrophil), bacterial distributions, results of drug sensitivity test and empirical antibiotic regimens. The 30-day mortality was used to evaluate the primary outcomes of the patients.

### Diagnosis criteria

The international classification of disease was employed to identify the diagnosis of cirrhosis and associated risk factors. The liver disease severity was confirmed by Child-Pugh score [38]. If the patients appeared one or more the following symptoms, the patients were considered to have an infection: while blood cells appearing in normally sterile body fluids; perforated viscus; pneumonia associated with purulent sputum demonstrated by radiographic imaging; some other symptoms caused by infection, such as ascending cholangitis [39]. Blood samples were collected each infected or seemingly infected case. Blood culture results of the patients were collected by following the standard operations. Gram-negative bacterial bloodstream infection was defined as the growth of any aerobic gram-negative bacteria in the blood culture. The patients who presented infection over 48h after admission to the hospital were defined as nosocomial infection. The center for disease control and prevention criteria were applied to confirm the primary source of BSI [40]. Whether SBP was a source of BSI was determined by the Fridman’s criteria [41]. The therapy was considered to be appropriate if the used drug could inhibit the activity of isolated pathogens *in vitro* according to drug sensitivity test. The definition of MDR was according to Magiorakos et al [42]. If the isolates show non-susceptibility to at least one agent in three or more antimicrobial categories *in vitro*, the isolates were defined as MDR.

### Statistical analysis

The continuous variables were presented as mean ± standard deviation (SD) and were compared by Student’s t test. The categorical data were analyzed by chi-square test. The clinical characteristics of included patients were compared based on their survival status within 30 days after infection diagnosis, infection acquisition sites, as well as the extended-spectrum β-lactamase (ESBL) status of their cultures. In addition, we also compared the clinical symptoms between the patients infected by MDR bacteria and those infected by non-MDR bacteria. Survival curves were calculated according to Kaplan-Meier method with log rank test. Cox regression analysis was used to identify the risk factors and independent indicators for 30-day mortality of the study subjects. SPSS 18.0 software was used for all statistical analyses and *P<* 0.05 was considered as statistical significance in the present study.
